# Biochemical Properties and Substrate Specificity of Two Acyl-CoA:Lysophosphatidic Acid Acyltransferases (*Pt*ATS2a and *Pt*ATS2b) from Diatom *Phaeodactylum tricornutum*

**DOI:** 10.3390/ijms26209936

**Published:** 2025-10-12

**Authors:** Katarzyna Jasieniecka-Gazarkiewicz, Ada Połońska, Yangmin Gong, Antoni Banaś

**Affiliations:** 1 Intercollegiate Faculty of Biotechnology, University of Gdansk and Medical University of Gdansk, 80-307 Gdansk, Poland; 2 Key Laboratory of Algal Biology, Institute of Hydrobiology, Chinese Academy of Sciences, Wuhan 430072, China

**Keywords:** LPAAT, diatoms, *Phaeodactylum tricornutum*, phosphatidic acid, *Pt*ATS2a, *Pt*ATS2b

## Abstract

Microsomal fractions from yeast Δ*ale1* cells harbouring the empty plasmid pYES2/CT and from yeast cells overexpressing *Pt*ATS2a (Phatr3_J11916) or *Pt*ATS2b (Phatr3_J43099) were used in the studies. When *sn*-1-18:1-LPA and [^14^C]16:0-CoA were used as exogenous substrates, both *Pt*ATS2a and *Pt*ATS2b showed the highest activity at 23 °C in the range of temperatures tested from 10 to 60 °C. Both enzymes showed the highest activity in alkaline pH. For *Pt*ATS2a, it was pH 10 while for *Pt*ATS2b, it was pH 11. At pH 6 and pH 12, the activities of both enzymes were very low. The calcium ions at concentrations of 0.05–1 mM drastically decreased the activity of both enzymes. The magnesium ions at a concentration of 0.05 mM had a little effect on the activity of both enzymes, while higher concentrations (0.5 mM and 1 mM) significantly inhibited their activity. To study the substrate specificity, seventeen different acyl-CoAs in combinations with *sn*-1-[^14^C]18:1-LPA were used. *Pt*ATS2a showed the highest preference for 18:4-CoA n-3 while *Pt*ATS2b for 18:1-CoA. The pattern of utilisation of other acyl-CoAs tested also differed between the two enzymes. The presented studies, for the first time, characterised LPAAT type enzymes from diatoms, organisms that naturally produced very-long-chain polyunsaturated fatty acids (VLC-PUFA).

## 1. Introduction

The biosynthesis of all glycerolipids starts with the acylation of position *sn*-1 of 3-phosphoglicerol with appropriate fatty acids, which is catalysed by acyl-CoA:3-phosphoglicerol acyltransferase (GPAT). The second acylation step is performed by enzymes with LPAAT (acyl-ACP/acyl-CoA:lysophosphatidic acid acyltransferase) activity. Phosphatidic acid (PA) synthesised in this reaction can be dephosphorylated by phosphatidic acid phosphatase, producing diacylglycerol (DAG). Both PA and DAG are then key precursors for the biosynthesis of all membrane glycerolipids and main storage lipids—triacylglycerols [1]. In higher plants, enzymes of LPAAT type are localised in plastids and in endoplasmic reticulum (ER). Generally, LPAAT localised in the plastids showed a preference towards palmitoyl-ACP (16:0-ACP) over other acyl-ACP (acyl-CoAs could also be utilised by these enzymes) and these, in the ER, show a preference for oleoyl-CoA over other acyl-CoAs as a fatty acids donors [1,2]. However, the substrate specificity of plant LPAATs towards acyl-CoAs can vary substantially among plant species [3,4,5,6,7]. At least two classes of ER-located LPAATs have been revealed: class A (ubiquitously present in the plant) exhibits preferences for 18:1-CoA and class B shows preferences for acyl-CoA with unusual acyl groups [5,6]. Except for several genes encoding LPAATs in higher plants, the gene encoding enzyme with such activity was also cloned from bacteria (*Escherichia coli*, *Nisseria meningitidis, Pseudomonas fluorescence*) and from yeast *Sacharomyces cerevisiae* [8,9]. Recently, genes encoding LPAAT-type enzymes were cloned from the green alga *Chlamydomonas reinhardtii* [10] and diatom *Phaeodactylum tricornutum* [11]. Enzymes with LPAAT activity are absolutely essential for cell growth. The enzymes with LPAAT activity seem to be present in cells with more than one isoform, encoded by different genes except in *E. coli*. The mutation of LPAAT encoding gene in *E. coli* (*plsC* gene) completely inhibited bacterial growth at higher temperatures; however, the mutation of one of the LPAAT encoding genes in *N. meningitidis* or *P. fluorescence* caused only some disturbances in their growth. Mutation of *slc1*—the gene encoding yeast LPAAT—does not have a significant effect on growth of yeast cells; its function could be overlapped by the *ale1* (named also *slc4*) gene, which encodes another yeast acyl-CoA:lysophospholipid acyltransferase. Knockout of both these genes is, however, lethal for yeast cells [8,9]. The mutation of gene encoding plastidial LPAAT in Arabidopsis is also lethal for such homozygous plants at an early stage of embryogenesis [12]. The mutation in gene encoding *Cr*LPAAT2—an ER-located LPAAT in Chlamydomonas, caused only a reduction in TAG content under nitrogen deprivation and did not affect the abundance of major glycerolipids [10]. The transcript abundance of Phatr3_J11916 (*PtATS2a*) or Phatr3_J43099 (*PtATS2b*), which encodes LPAATs in *P. tricornutum*, affected cell growth rate as well as membrane and storage lipid content and composition [11]. Except for the study mentioned above with Arabidopsis plants, there are no other comprehensive studies of the effect of knocking out genes encoding LPAAT-type enzymes in other higher plants.

Contrary to the higher plants, LPAATs from microalgae have received no detailed characterisation. Concerning the diatom *P. tricornutum* LPAATs, in a previously published paper [11], only the LPAAT activity of the enzymes encoded by *PtATS2a* and *PtATS2b* has been shown using assays with different lysophospholipids and 16:0-CoA or 18:1-CoA. Here, we report the detailed biochemical characterisation of these *Pt*LPAATs (pH, temperature and selected ions dependency) as well as their substrate specificity towards a broad set of acyl-CoAs. These results will complement the data of the mutation effect of *PtATS2a* or *PtATS2b* genes [11] on the physiology of *P. tricornutum* cells. The presented biochemical characterisations of *Pt*ATS2a and *Pt*ATS2b, especially their preferences towards different acyl-CoA, could also be useful for plant biotechnologists. Currently, considerable efforts are being made to transfer the machinery of biosynthesis of VLC-PUFA from microorganisms to higher plants. The genes of *P. tricornutum*, an organism that naturally produces such fatty acids, could be a potential source for such modification of higher plants, and biochemical characterisation of the products of these genes will facilitate their proper utilisations.

## 2. Results

### 2.1. Effect of Environmental Factors on the Activity of P. tricornutum Two Enzymes with LPAAT Activity

Among the environmental factors that potentially affect the tested enzymes’ activity are temperature, pH, and different ions (especially divalent cations). In the present study, the effects of all of these factors on *Pt*ATS2a and *Pt*ATS2b (enzymes of *P. tricornutum* with LPAAT activity) were studied.

**Figure 1:**
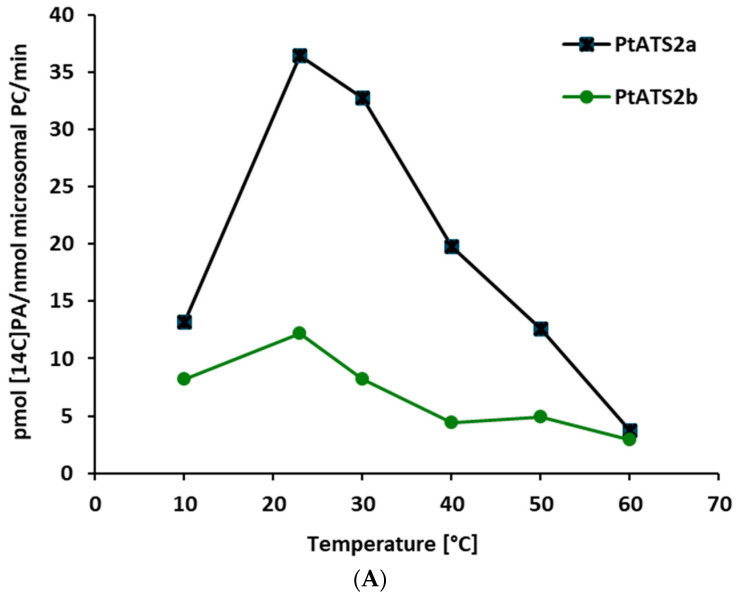

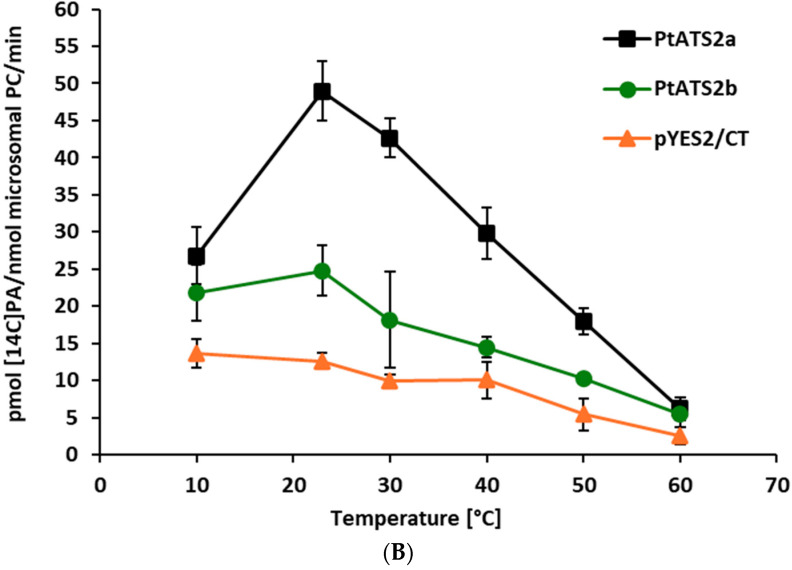
(**A**) Effect of temperature on the LPAAT activity of *Pt*ATS2a and *Pt*ATS2b encoded, respectively, by Phatr3_J11916 and Phatr3_J43099 genes of *P. tricornutum*. Differences in mean activities obtained in assays with microsomes of yeast Δ*ale1* mutant carrying the tested enzymes and the mean control activity (yeast Δ*ale1* mutant transformed with an empty plasmid) are presented. Mean activities with standard deviations are presented in (**B**). (**B**) Effect of temperature on LPAAT activity in control yeast microsomal fractions (yeast Δ*ale1* mutant transformed with an empty plasmid pYES2/CT) and in microsomal fractions of yeast Δ*ale1* mutant overexpressing Phatr3_J11916 (encoding *Pt*ATS2a) and Phatr3_J43099 (encoding *Pt*ATS2b) genes of diatom *P. tricornutum*. Mean values and SD are presented (data from at least three independent assays).

The effect of the temperature on *Pt*ATS2a and *Pt*ATS2b activity was examined in the range from 10 to 60 °C. Of these two enzymes, *Pt*ATS2a showed a stronger response to the changes in temperature. Its activity increased by approximately 2.8 times when the temperature changed from 10 °C to 23 °C and reached its maximum value at this temperature. Further increases in temperature to 30 °C, 40 °C, 50 °C, and 60 °C caused a gradual decrease in the *Pt*ATS2a activity by approximately 14, 49, 68, and 86%, respectively, with the highest activity recording at 23 °C. The activity of *Pt*ATS2b changed in the temperature range from 10 °C to 60 °C in a somewhat similar way to the activity of *Pt*ATS2a. The main difference was a less drastic increase in the activity of *Pt*ATS2b in the temperature range of 10 °C to 23 °C (about 50% increases in activity). The results are presented in Figure 1A,B. In the first figure, the activity assigned only to the tested enzymes is presented, and in the second figure, the overall activity obtained in the assays with microsomal fractions of the yeast carrying the tested enzymes (i.e., the sum of background LPAAT activity and the activity of tested enzyme) as well as the background LPAAT activity only is shown.

**Figure 2:**
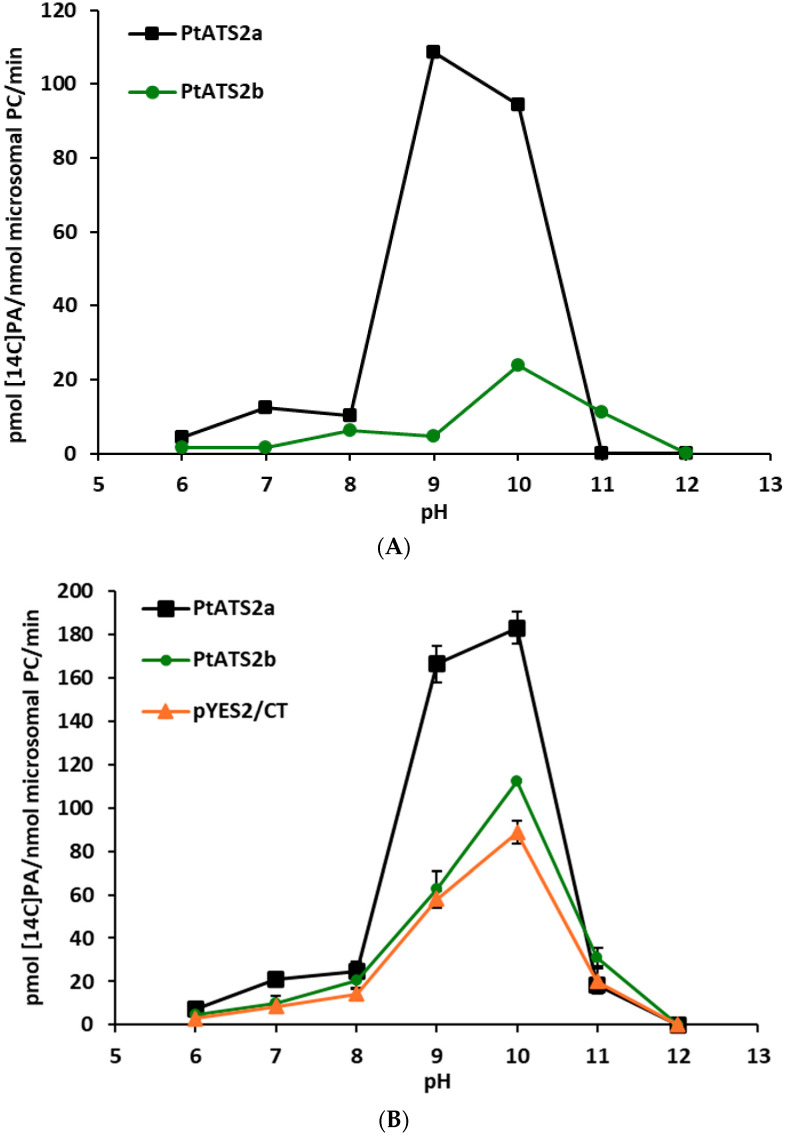
(**A**) Effect of pH on the LPAAT activity of *Pt*ATS2a and *Pt*ATS2b encoded, respectively, by Phatr3_J11916 and Phatr3_J43099 genes of *P. tricornutum*. Differences in mean activities obtained in assays with microsomes of yeast Δ*ale1* mutant carrying the tested enzymes and the mean control activity (yeast Δ*ale1* mutant transformed with an empty plasmid) are presented. Mean activities with standard deviations are presented in (**B**). (**B**) Effect of pH on LPAAT activity in control yeast microsomal fractions (yeast Δ*ale1* mutant transformed with an empty plasmid pYES2) and in microsomal fractions of yeast Δ*ale1* mutant overexpressing Phatr3_J11916 (encoding *Pt*ATS2a) and Phatr3_J43099 (encoding *Pt*ATS2b) genes of diatom *P. tricornutum*. Three buffers with different pH ranges were used: 0.04 M phosphate buffer—pH 6.0–7.0, 0.04 M Tris-HCl buffer—pH 8.0–10.0, and 0.04 M Na_2_HPO_4_-NaOH buffer—pH 11.0–12.0. Mean values and SD are presented (data from at least three independent assays).

Another external factor tested was the pH of the incubation buffers. Assays were performed with four different buffers: 0.04 M phosphate buffer (pH 6.0–7.0), 0.04 M Tris-HCl (pH 8.0–10.0), 0.04 M NaHCO_3_-NaOH (pH 11.0), and 0.04 M Na_2_HPO_4_-NaOH (pH 12.0). *P*tATS2a had very low activity at pH 6.0. This activity increased with the increase in pH to 7.0 (first peak of activity) and dropped somewhat down at the pH of 8.0. Further increase in pH to 9.0 caused a rapid increase in the *Pt*ATS2a activity (about ten times; a second peak of activity). At pH 10.0, a decrease in activity of about 13% in comparison to the maximum one (from pH 9.0) was recorded. Then, *Pt*ATS2a started to lose its activity rapidly, and at pH 11.0 and 12.0, its activity was close to zero. The second enzyme studied, *Pt*ATS2b, also had very low activity at pH 6.0. The activity of this enzyme remained at a very low level also at pH 7.0. At pH 8.0, the activity of *Pt*ATS2b was increased (about 5.5 times higher than the activity at pH 6.0; first peak of activity) and, at pH 9.0, somewhat dropped down. The second peak of activity of *Pt*ATS2b was recorded at pH 10. At pH 11.0, *Pt*ATS2b lost about half of activity recorded at pH 10 and at pH 12.0, it did not show LPAAT activity. The response pattern of the *Pt*ATS2b activity to pH values of incubation media was somewhat similar to that of *Pt*ATS2a, with a shift in first peak of activity from pH 7 to pH 8 and the second peak of activity from pH 9 to pH 10. Furthermore, its activity was generally lower than that of *Pt*ATS2a. Results are presented in Figure 2A,B (activity assigned only to the tested enzymes and overall activity obtained in the assays with tested microsomal fractions, respectively).

**Figure 3:**
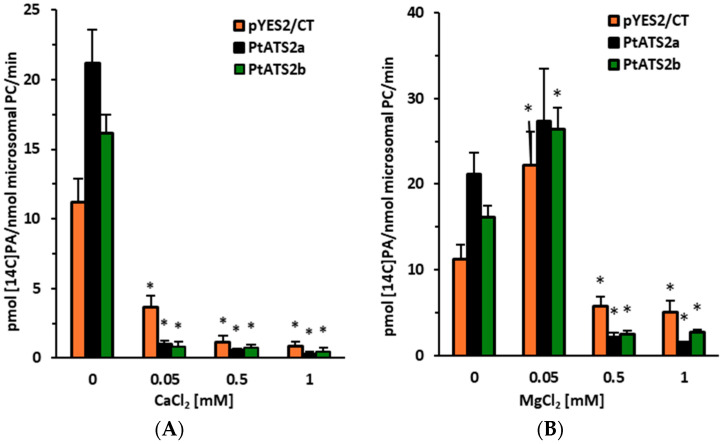
Effect of Ca^2+^ (**A**) and Mg^2+^ (**B**) ions on the LPAAT activity in control yeast microsomal fractions (yeast Δ*ale1* mutant transformed with an empty plasmid pYES2) and in microsomal fractions of yeast Δ*ale1* mutant overexpressing Phatr3_J11916 (encoding *P*tATS2a) and Phatr3_J43099 (encoding *Pt*ATS2b) genes of *P. tricornutum*. Mean values and standard deviations (SDs) are presented (data from at least three independent assays). Asterisks indicate statistical significance of the LPAAT activities between the control (ions not added) and the tested ions’ concentrations in assayed microsomal fractions, as determined by Student’s *t* test (*p* ≤ 0.05).

The last environmental factor studied on *Pt*ATS2a and *Pt*ATS2b activity was the calcium or magnesium ion present in the reaction buffer. For this type of assays, 0.04 M HEPES buffer (pH 7.2) was used due to the poor solubility of magnesium and calcium ions in phosphate buffer. Calcium ions (in the form of CaCl_2_) in the range of concentration 0.05–1 mM strongly inhibited the activity of both tested enzymes. The background LPAAT activity (caused by yeast endogenous SLC1 enzyme) was also inhibited, however, to a somewhat lower extent (Figure 3A). Lower LPAAT activity in assays with microsomal fractions of yeast overexpressing the tested enzymes (in the presence of Ca^2+^) than in assays with microsomal fractions of yeast transformed only with the empty plasmid; this could suggest that the overexpression of the tested enzymes reduced the amount/activity of the endogenous SLC1. The magnesium ions (in form of MgCl_2_; used in the same concentrations as calcium ions) clearly inhibited the LPAAT activity of the tested enzymes at concentration (in incubation buffers) 0.5 and 1 mM. The 0.05 mM concentration of Mg^2+^ probably also had some inhibitory effect on LPAAT activity of *Pt*ATS2a and *Pt*ATS2b; the differences between these enzymes’ activity and background activity in the presence of magnesium ions in that concentration was lower than in assays without magnesium ions. However, due to the stimulation of activity of yeast endogenous SLC1 by this magnesium concentration, the inhibition was less visible (Figure 3B).

### 2.2. Substrate Specificity of PtATS2a and PtATS2b Towards Different Acyl-CoAs

In this study, seventeen different fatty acid donors, i.e., thioesters of these fatty acids with coenzyme A (acyl-CoA), in combinations with *sn*-1-[^14^C]18:1-LPA, were used.

**Figure 4:**
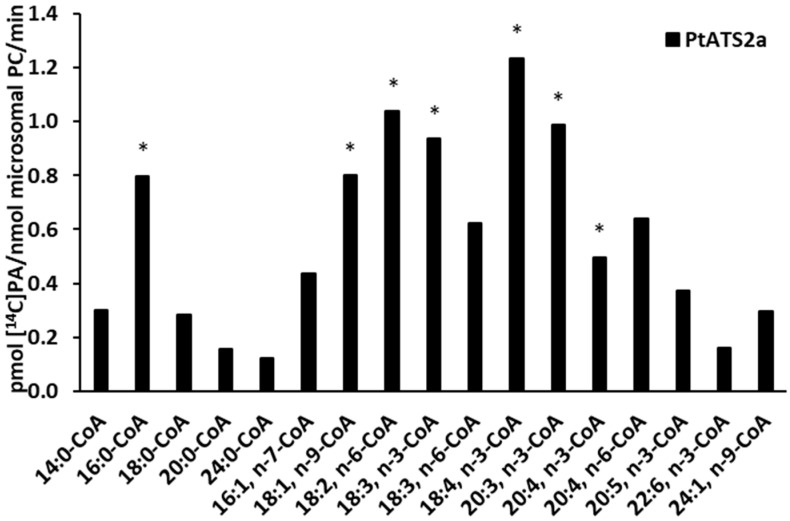
LPAAT activity of *Pt*ATS2a (a diatom *P. tricornutum* enzyme encoded by the Phatr3_J11916 gene) in assays with [^14^C]18:1-LPA as acyl acceptor and 17 different acyl-CoAs (tested separately) as fatty acids donors. Differences in mean activities obtained in assays with microsomes of yeast Δ*ale1* mutant expressing the tested enzymes and the mean control activity (yeast Δ*ale1* mutant transformed with an empty plasmid) are presented. Asterisks indicate statistical significance of LPAAT activity between the control microsomes and microsomes carrying *Pt*ATS2a, as determined by Student’s *t* test (*p* ≤ 0.05). Mean activities with standard deviations are presented in Figure 6.

*Pt*ATS2a showed the highest preference for 18:4-CoA n-3. The preferences of *Pt*ATS2a towards other acyl-CoAs tested, from the best to the worst utilised, were as follows: 18:2-CoA n-6, 20:3-CoA n-3, 18:3-CoA n-3, 18:1-CoA n-9, 16:0-CoA, 20:4-CoA n-6, 18:3-CoA n-6, 20:4-CoA n-3, 16:1-CoA n-7, 20:5-CoA n-3, 14:0-CoA, 24:1-CoA, 18:0-CoA, 22:6-CoA n-3, 20:0-CoA, and 24:0-CoA. The activity of the tested enzyme relative to 18:2-CoA n-6, 20:3-CoA n-3, and 18:3-CoA n-3 oscillated between 84% and 76% of the highest activity noted in relation to 18:4-CoA n-3. In assays with 18:1-CoA n-9, 16:0-CoA, 20:4-CoA n-6, 18:3-CoA n-6, 20:4-CoA n-3, 16:1-CoA n-7, and 20:5-CoA n-3, the observed activity was between 65% and 30% of the highest one. The lowest preference of *Pt*ATS2a was noted for 14:0-CoA, 24:1-CoA, 18:0-CoA, 22:6-CoA n-3, 20:0-CoA, and 24:0-CoA. The activity of *Pt*ATS2a in assays with these acyl-CoAs ranged between 24% and 10% of the highest activity noted in assays with 18:4-CoA n-3 (Figure 4).

**Figure 5:**
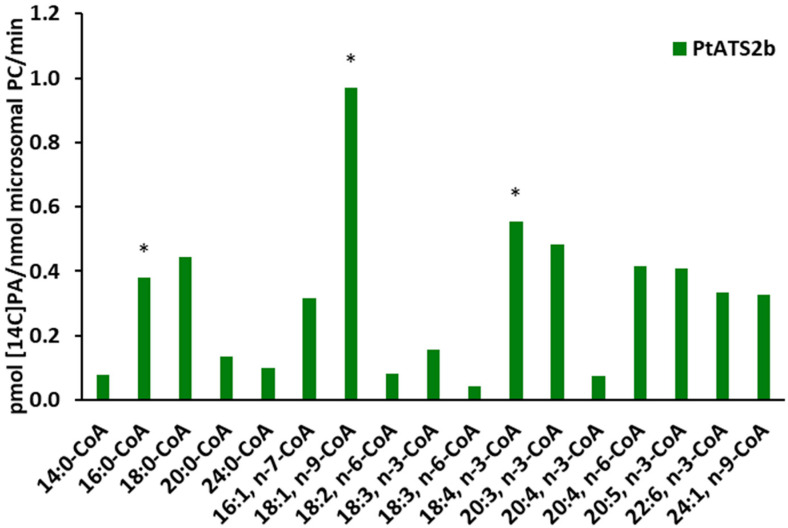
LPAAT activity of *Pt*ATS2b (the diatom *P. tricornutum* enzyme encoded by the Phatr3_J43099 gene) in assays with [^14^C]18:1-LPA as acyl acceptor and 17 different acyl-CoAs (tested separately) as fatty acids donors. Differences in mean activities obtained in assays with microsomes of yeast Δ*ale1* mutant carrying the tested enzyme and the mean control activity (yeast Δ*ale1* mutant transformed with an empty plasmid) are presented. Asterisks indicate statistical significance of LPAAT activity between the control microsomes and microsomes carrying *Pt*ATS2b, as determined by Student’s *t* test (*p* ≤ 0.05). Mean activities with standard deviations are presented in Figure 6.

**Figure 6:**
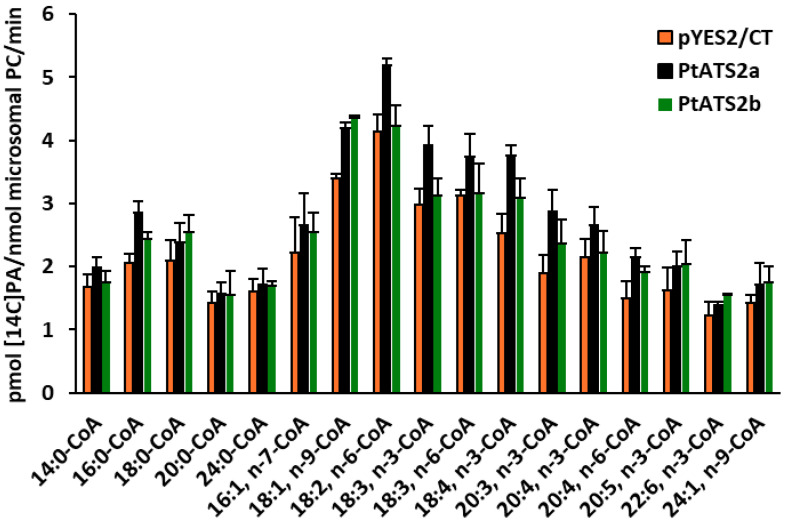
LPAAT activity in control yeast microsomal fractions (yeast Δ*ale1* mutant transformed with an empty plasmid pYES2/CT) and in microsomal fractions of yeast Δ*ale1* mutant overexpressing Phatr3_J11916 (encoding *Pt*ATS2a) and Phatr3_J43099 (encoding *Pt*ATS2b) genes of diatom *P. tricornutum.* Mean values and SD are presented (data from at least three independent assays).

The second enzyme, *Pt*ATS2b, also showed a broad acceptance of tested acyl-CoAs; however, its preferences differed from those of *Pt*ATS2a. The best accepted acyl-CoA by *Pt*ATS2b was 18:1-CoA n-9. The subsequent acyl-CoAs (listed in decreasing order of acyl-CoA preference) accepted by *Pt*ATS2b were 18:4-CoA n-3, 20:3-CoA n-3, 18:0-CoA, 20:4-CoA n-6, 20:5-CoA n-3, 16:0-CoA, 22:6-CoA n-3, 24:1-CoA, 16:1-CoA n-7, 18:3-CoA n-3, 20:0-CoA, 24:0-CoA, 18:2-CoA n-6, 14:0-CoA, 20:4-CoA n-3, and 18:3-CoA n-6. The observed activity of *Pt*ATS2b towards the first nine from the presented list ranged between 57% and 33% of the highest activity. The preference for the last seven acyl-CoAs from the presented list ranged between 16% and 5% of the activity noted for 18:1-CoA n-9 (Figure 5). The increase in LPAAT activity over the background activity in assays with microsomal fraction of yeast overexpressing the tested enzymes was not always statistically proven (Figure 4, Figure 5 and Figure 6). However, the background LPAAT activity in these assays could be potentially lower than that in control microsomal fraction (yeast transformed with empty vector) as the assays suggested with divalent cations described above. Thus, the real LPAAT activity of the tested enzymes could be higher, and even if was not statistically significant, it could be a real activity attributed to these enzymes. The greatest differences in preferences for fatty acid donors (acyl-CoA) between *Pt*ATS2a and *Pt*ATS2b enzymes concerned such acyl-CaAs as the following: 18:2-CoA n-6, 18:3-CoA n-3, 18:3-CoA n-6 and 20:4-CoA n-6. The activity of the *Pt*ATS2b enzyme towards these fatty acid donors was only about 7–17% of the activity of the *Pt*ATS2a enzyme. The activity of these two enzymes was similar towards 20:5-CoA n-3, 24:1-CoA n-9, 20:0-CoA, 24:0-CoA (listed in order from most to least preferred).

## 3. Discussion

In research characterising biochemical properties and/or substrate specificities of acyl-CoA:lysophospholipid acyltransferases (LPLATs), yeast *Saccharomyces cerevisiae* with a mutated *ale1* gene is used most often as a host for heterologous expression of studied gene/enzyme. *S. cerevisiae* cells contain two main types of LPLAT enzymes; ALE1 (the most active) with high specificity towards lysophosphatidylcholine (LPC) and lysophosphatidyletanolamine (LPE), and SLC1 with highest specificity towards lysophosphatidic acid (LPA) (for references, see Introduction). The *S. cerevisiae* Δ*ale1* mutant overexpressed with the studied gene/enzyme also exhibit, except for the LPLAT type of activity of introduced enzyme, the LPLAT activity of yeast endogenous SLC1. Thus, in assays characterising the studied LPLAT enzyme (present in microsomal fraction of overespressor), the background activity (caused by SLC1) is treated as the LPLAT activity from assays performed in parallel with microsomes of yeast transformed with an empty plasmid.

Using such a yeast system as described above, in this work, we characterised the biochemical properties and substrate specificity of two enzymes of LPAAT type (*Pt*ATS2a and *Pt*ATS2b) encoded, respectively, by Phatr3_J11916 and Phatr3_J43099 genes derived from *P. tricornutum* [11]. Both tested enzymes showed some similarity to LPAAT enzymes that have been tested so far; however, they also possessed unique features characteristic only to each of them.

Both *Pt*ATS2a and *Pt*ATS2b showed the highest activity at 23 °C. However, *Pt*ATS2a was more temperature-dependent than *Pt*ATS2b. Its activity varied more than the activity of *Pt*ATS2b, especially during the raise of the temperature from 10 to 23 °C (for *Pt*ATS2a, it was 2.8-fold increase in activity, and for *Pt*ATS2b, it was about a 0.5-fold increase in activity).

The optimal temperature for *P. tricornutum* growth is estimated to be 15–21 °C [13,14]. In that range of the environmental temperatures, the activity of *Pt*ATS2a and *Pt*ATS2b are also almost optimal. Thus, the optimal temperature for their activity is well correlated with the optimal temperature for *P. tricornutum* development.

The response of *Pt*ATS2a and *Pt*ATS2b to ambient temperature differed from that of *C. sativa* seed LPAATs [7]. The most significant differences concerned the activity at a lower range of the tested temperatures. The *C. sativa* LPAATs at 10 °C were more than 20 times less active than at 30 °C, where their maximum activity was annotated. In contrast to *C. sativa* LPAATs, endogenous yeast LPAAT—SLC1—was most active at the range of temperature from 10 to 20 °C (Figure 2B); thus, the dependency of its activity to the change in temperature was a bit similar to the tested *P. tricornutum* LPAATs. At 60 °C, the activity of LPAATs of all three discussed species were very low (close to detection level). From a biotechnological point of view, the introduction of the genes encoding *Pt*ATS2a or *Pt*ATS2b to the potential transgenic crops will not only modify the substrate specificity of their LPAAT but could also increase the LPAAT activity, especially at temperatures lower than 20 °C. This could be especially important for crops cultivated in cold and moderate climates.

The response of *Pt*ATS2a and *Pt*ATS2b to changes in environmental pH was somewhat similar, however, not the same. Both enzymes showed two peaks of activity; in neutral pH (small one) and in alkaline pH (the highest activity). The first peak of activity for *Pt*ATS2a was at pH 7, and the second one at pH 10, while for *Pt*ATS2b, it was at pH 8 and pH 11, respectively. Thus, both peaks of activity of *Pt*ATS2b were shifted towards more alkaline pH compared to *Pt*ATS2a. At pH 11, *Pt*ATS2a showed virtually no enzymatic activity, whereas *Pt*ATS2b lost this activity only at pH 12. At pH 6, the activity of both enzymes was very low.

Some of the plant LPAATs tested so far also showed high activity at alkaline pH. Ichihara et al. [15] demonstrated that LPAATs derived from safflower (*C. tinctorius*) seeds showed the maximum activity in the pH range of 8.5–9.5, with high activity also at pH 7 and 10. Oo and Huang [3] in their studies on rapeseed (*B. napus*) LPAATs noted their optimum pH above 10.0, with rather high activity also between pH 6–10. The highest activity of *Camelina sativa* seeds LPAATs was at pH 7.5 to pH 9.0, but with high activity also at pH 7 and 10 [7]. However, palm (*Syagrus cocoides*) and maize (*Zea mays*) seeds LPAATs showed the highest activity at pH 6–7 and a second small peak of activity at alkaline pH (10–11) [3]. Studied during the course of presented research, endogenous yeast LPAAT (SLC1) showed a gradual increase in activity between pH 6–8, a sharp increase in its activity between pH 8–10, and a sharp decrease in activity in more alkaline pH (Figure 3B). Thus, it seems that there is no one pattern of response of LPAATs from different organisms (or even from plants) to environmental pH. Each of the so far tested LPAATs showed their own unique pattern of shifts in activity in response to changes in pH.

The alkaline pH at which the highest activity of the tested enzymes was annotated was not used, however, in other assays. The most probable pH inside cells’ cytosol is around pH 7. Thus, in all other tests, we used pH 7.2. Nevertheless, in certain cellular compartments, pH can differ from cytosolic pH.

From the environmental factors which potentially affect *Pt*ATS2a and *Pt*ATS2b activity, the effect of magnesium and calcium ions was also studied. The calcium ions at concentrations of 0.05–1 mM drastically decreased the activity of both enzymes. However, the effect of magnesium ions was somewhat different. The lowest concentration of magnesium ions used (0.05 mM) had only a minor effect on the activity of both enzymes, while higher concentrations (0.5 mM and 1 mM) significantly inhibited their activity. An inhibitory effect of calcium ions was also noted for LPAAT enzymes from palm (*S. cocoides*), corn (*Z. mays*), and rapeseed (*B. napus*), [3]. A stimulating effect of magnesium ions (at a concentration of 1 mM) was noted for LPAAT enzymes from corn and palm [3]. The same authors also noted that magnesium ions at that concentration had an inhibitory effect on LPAATs derived from rapeseed. In the study presented by Cao et al. [16], magnesium ions were found to have a stimulating effect on the activity of LPAATs derived from *Limnanthes alba*. Endogenous yeast LPAAT (SLC1) responded in a similar manner to the tested *P. tricornutum* LPAATs. The tested calcium concentrations inhibited its activity; however, magnesium ions at the lowest concentration stimulated its activity, and in higher concentrations, clearly inhibited the activity. Thus, LPAATs from different organisms react in a similar manner to calcium and magnesium ions; calcium ions always inhibit their activity (at least in the tested concentrations), and magnesium ions could have a stimulatory, neutral or inhibitory effect depending on the concentrations used in the assay.

Tests on the substrate specificity of *Pt*ATS2a and *Pt*ATS2b for various acyl-CoAs showed that these enzymes exhibited different preferences for different “species” of these fatty acid donors. *Pt*ATS2a showed the highest preference for 18:4-CoA n-3, while *Pt*ATS2b for 18:1-CoA, followed by 18:4-CoA n-3. Furthermore, *Pt*ATS2a exhibited significantly higher activity towards 18:2-CoA n-6, 18:3-CoA n-3, and 18:3-CoA n-6 than *Pt*ATS2b. Both enzymes also accepted the remaining fatty acids from the EPA biosynthetic pathway (apart from the mentioned 18:4-CoA) such as 20:4 n-3, 20:4 n-6, and 20:5 n-3. However, higher activity of these enzymes can be observed for 20:4 n-6 than for 20:4 n-3. As discussed in our earlier paper regarding *Pt*LPCAT1 [17], the activity toward 20:4-CoA n-3 may be important during the biosynthesis of 20:5 (EPA). However, the influence of *Pt*LPAAT enzymes on the EPA biosynthesis process can be considered only as an indirect effect. The PA molecules they synthesise could be a supplier of DAG for PC biosynthesis, and the fatty acid composition of such synthesised PC molecules could be further modified, providing EPA or other intermediates from its biosynthetic pathway in the case of fatty acids other than 20:4, which are subject to modification. In the study presented by Klińska et al. [7], LPAATs derived from *C. sativa* seeds showed a preference for 18-carbon unsaturated fatty acids, especially to 18:1. Among saturated fatty acids, they accepted 16:0 relatively well. LPAATs from *P. tricornutum* also showed high activity towards 16:0-CoA and 18:1-CoA. As for the remaining 18-carbon polyunsaturated fatty acids, *Pt*ATS2a accepted them relatively well, while *Pt*ATS2b accepted them relatively poorly. In the study conducted by Klińska et al. [7], the preference of plant LPAATs towards acyl-CoA with fatty acids longer than 18-carbon atoms or with more than three double bonds was not tested. Therefore, the specificity of LPAATs from *P. tricornutum*, which accepted many of these fatty acids relatively well, cannot be compared with them. However, based on studies on the substrate specificity of *P. tricornutum* LPAATs (*Pt*ATS2a and *Pt*ATS2b), it can be assumed that each of them has its own unique substrate specificity.

The mutation of *Pt*ATS2a or *Pt*ATS2b significantly affected *P. tricornutum* growth and lipid contents in the cells of this diatom [11]. The growth retardation and the changes in lipid content and composition caused by inactivation of *Pt*ATS2a were not the same as that caused by inactivation of *Pt*ATS2b. Moreover, the cells’ response to the mutation of the mentioned genes was different in different growth environments (nitrogen-replete and nitrogen-free media). In the presented work, we have shown the differences in biochemical properties and substrate specificities of both tested *P. tricornutum* enzymes; however, it is difficult to precisely connect these differences with the observed effects of their mutations. Moreover, both enzymes have distinct localisation in diatom chloroplast systems, which could additionally influence the defects appearing after their mutation.

From a biotechnological point of view, especially in plant modification towards biosynthesis of VLC-PUFA, *Pt*ATS2a characterised better substrate specificity. It accepted a broader spectrum of fatty acids from the biosynthetic pathway of these fatty acids than *Pt*ATS2b. However, only their introduction into oilseed crops can demonstrate their usefulness in production of VLC-PUFA by these plants.

## 4. Materials and Methods

### 4.1. Chemicals

Non-radiolabelled and ^14^C labelled acyl-CoA were synthesised in our laboratory according to modified method described by Sanchez et al. [18]. ^14^C-labelled fatty acids and *sn*-1-[^14^C]18:1-LPA were purchased from PerkinElmer Life Science (Waltham, MA, USA). Non labelled fatty acids were purchased from (Avanti Polar Lipids, Alabaster, AL, USA). Other chemicals were purchased from (Merck, Darmstadt, Germany) unless another company was indicated.

### 4.2. Yeast Strains

The yeast *Saccharomyces cerevisiae* Δ*ale1* cells, with the introduced plasmid pYES2/CT or plasmid pYES2/CT harbouring *Phaeodactylum tricornutum* LPAAT encoding gene (*Pt*ATS2a or *Pt*ATS2b) were used in the experiments. *S. cerevisiae* haploid knockout mutant Δ*ale1* (*Y02431*; Matα; his3∆1; leu2∆0; lys2∆0; ura3∆0; YOR175c::kanMX4) was transformed either with the pYES2/CT plasmid carrying one of the two genes: Phatr3_J11916, or Phatr3_J43099, under the control of GAL1 promoter, or with the empty pYES2/CT plasmid (control). A detailed methods section, including plasmid construction and *Pt*ATS2 mutants is presented in Supporting Information Methods S1 in our previous work [11].

### 4.3. Yeast Cultivation and Isolation of Microsomal Fractions

Yeast cells were cultured for 24 h with shaking (220 rpm) at 30 °C in synthetic uracil dropout medium containing 2% glucose. After that time, galactose was added (final concentration of galactose in the medium was 2%) and the cells were grown for an additionally for 24 h. After that time, yeast cells (OD reached 3–4) were harvested by centrifugation (Rotofix 32 A centrifuge; Hettich, Tuttlingen, Germany) at 3600 rpm for 10 min. The obtained pellets were washed twice with 50 mL distilled water. After the second centrifugation, the washed pellets were suspended in “glass bead disruption buffer” (20 mM Tris-HCL—pH 7.9, 10 mM MgCl_2_, 1 mM EDTA, 5% glycerol, 0.3 M ammonium sulphate) supplemented with protease inhibitors (Roche, Basel, Switzerland). Yeast suspensions were transferred to 2 mL plastic tubes with screws, filled to ¾ of their volume with glass beads (0.45–0.5 mm in diameter). The tubes were shaken ten times for 30 s in Mini Bead Beater (BioSpec Products, Bartesville, OK, USA). The crushed yeast cells with glass beads were transferred to 50 mL plastic tubes and centrifuged (Rotofix 32 A centrifuge; Hettich) in a cold room for 10 min at 4000 rpm. The resulting supernatants were filtered through Miracloth into centrifuge tubes, which were subsequently centrifuged for two hours at 42,000 rpm (100,000× *g*) in a L-70 ultracentrifuge (Beckman, Brea, CA, USA). The pellets formed after centrifugation, containing microsomal fractions, were washed briefly with phosphate buffer (0.1 M; pH 7.2) and then suspended in a small amount of this buffer (modified method according to Dahlqvist et al. [19]). The obtained microsomal fractions were stored at −80 °C for further analysis. Aliquots of the obtained microsomal fractions were used for phosphatidylcholine content determination. Its concentration in the microsomes was determined by performing lipid extraction using modified Bligh and Dyer [20] method, separating lipids of chloroform fractions by thin-layer chromatography, and measuring the fatty acid content of the isolated phosphatidylcholine (PC) by gas chromatography analysis (details in Ref. [7]). Aliquots of microsomal fractions were also used for total protein determination. Protein concentration was determined according to the manufacturer’s instructions for the BCA Assay Kit (Pierce Chemical, Dallas, TX, USA).

### 4.4. Enzyme Assay

**Figure 7:**
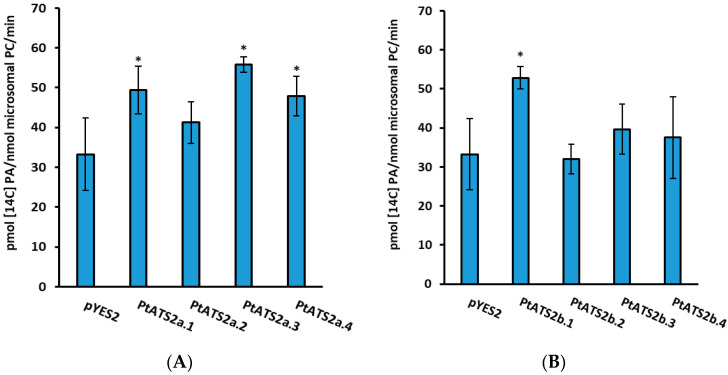
The *Pt*ATS2a (panel (**A**)) and *Pt*ATS2b (panel (**B**)) activity in control yeast microsomal fractions (yeast Δ*ale1* mutant transformed with an empty plasmid pYES2/CT) and in microsomal fractions of yeast overexpressing Phatr3_J11916 (*Pt*ATS2a) or yeast overexpressing Phatr3_J43099 *Pt*ATS2b). In the case of control yeast, the average value from four transformation events and in the case of Phatr3_J11916 or Phatr3_J43099 overexpressors, the data for each transformation event are presented. [^14^C]18:1-CoA together with *sn*-1–18:1-LPA were used as acyl donor and acceptor, respectively. Mean values and SD are presented. Asterisk denotes significant differences between control and Phatr3_J11916 or Phatr3_J43099 overexpressors, in “mean difference two-sided test” at α = 0.05.

Aliquots of microsomal fractions contained 0.5 nmol microsomal PC (equivalent to 2.2 µg of microsomal protein) was used as a source of tested enzymes in all assays. For microsomal preparation of yeast transformed with tested genes, the colonies expressed the highest LPAAT activity were selected. In the case of yeast transformed with Phatr3_J11916 gene (encoding *Pt*ATS2a) it was yeast from transformation event “3”, and for yeast transformed with Phatr3_J43099 gene (encoding *Pt*ATS2b), it was yeast from transformation event “1” [11] (Figure 7). As a control (background LPAAT activity), microsomal fractions from yeast transformed with empty pYES2/CT plasmid were used.

In assays to determine the effects of temperature, pH, and divalent ions (Mg^2+^ and Ca^2+^) on tested enzyme activities, 5 nmol of [^14^C]16:0-CoA (acyl donor) and 5 nmol of *sn*-1-18:1-LPA (acyl acceptor) were used as enzymes substrates. Assays measuring the substrate specificity towards acyl donors were performed with 3 nmol of *sn*-1-[^14^C]18:1-LPA in combination with 5 nmol of each of the seventeen non-radioactive acyl-CoA. The tested acyl-CoA was as follows: 14:0-CoA, 16:0-CoA, 18:0-CoA, 20:0-CoA, 24:0-CoA, 16:1^Δ9^-CoA, 18:1^Δ9^-CoA, 18:2^Δ9,12^-CoA, 18:3^Δ9,12,15^-CoA, 18:3^Δ6,9,12^-CoA, 18:4^Δ6,9,12,15^-CoA, 20:3^Δ11,14,17^-CoA, 20:4^Δ8,11,14,17^-CoA, 20:4^Δ5,8,11,14^-CoA, 20:5^Δ5,8,11,14,17^-CoA, 22:6^Δ4,7,10,13,16,19^-CoA, 24:1^Δ15^-CoA, 26:0-CoA.

The reactions were carried out in Eppendorf tubes with 0.04 M potassium phosphate buffer (pH 7.2; 100 µL), with two exceptions. The first concerned assays aimed at estimating the effects of pH on enzyme activity (the used buffers are presented in legend of Figure 2B). The second concerned assays measuring the effect of Mg^2+^ and Ca^2+^ ions on tested *Pt*LPAATs activity, where HEPES buffer (0.04 M; pH 7.2) was used (the tested ions in potassium phosphate buffer could form insoluble salts).

Assays were incubated for 60 min at 23 °C (except for the measurement the effect of tested temperature on enzyme activity) in Eppendorf Thermomixer Compact with continuous shaking at 1250 rpm. Enzymatic reactions were terminated by addition of 375 µL of chloroform/methanol (1:2; *v*:*v*), 5 µL of glacial acetic acid, 125 µL of chloroform and 125 µL of water. The samples were mixed vigorously and centrifuged to separate bottom chloroform layer from upper methanol–water layer. Chloroform fractions containing lipids were collected and separated by thin-layer chromatography on silica gel 60 plates from Merck using a polar solvent system consisting of chloroform/methanol/acetic acid/water (90:15:10:2.5; *v*:*v*:*v*:*v*). [^14^C]PA products were visualised, identified on the basis of the used ^14^C-labelled standards, and quantitated using an autoradiograph (Instant Imager; Canberra Packard, Schwadorf, Austria).

### 4.5. Presentation of the Activity of Tested Enzymes

The activity of the tested enzymes (PtATS2a and PtATS2b) is presented as the differences in LPAAT activity in assays with microsomal fractions of yeast overexpressing tested enzyme and microsomal fractions of yeast transformed with an empty plasmid. By such calculation, we assumed that endogenous LPAAT activity caused by the yeast SLC1 enzyme (of LPAAT type) is the same in both type of microsomes/assays. However, some of our results (for instance, presented experiments evaluating the effects of divalent cations on the activity of tested enzymes) indicated that overexpression of the studied genes can reduce somewhat-endogenous LPAAT activity. Unfortunately, there is no way to precisely measure the LPAAT activity caused by SLC1 and the part of LPAAT activity caused by introduced PtATS2a or PtATS2b. Thus, the LPAAT activity of tested enzymes presented in this way has to be treated as approximate activity, probably slightly lower than the real one. However, we think that this is the best way of presentation/measurement of the activity of studied enzymes. In the presented results, we are also showing the original data obtained in performed assays.

## Data Availability

Dataset available on request from the authors.
